# Fibulin1C peptide induces cell attachment and extracellular matrix deposition in lung fibroblasts

**DOI:** 10.1038/srep09496

**Published:** 2015-04-02

**Authors:** Qi Ge, Ling Chen, Jade Jaffar, William Scott Argraves, Waleed O. Twal, Phil Hansbro, Judith L. Black, Janette K. Burgess, Brian Oliver

**Affiliations:** 1 Respiratory Cellular and Molecular Biology Group, Woolcock Institute of Medical Research, Sydney, NSW 2037, Australia; 2 Discipline of Pharmacology, Sydney Medical School, The University of Sydney, NSW 2006, Australia; 3 Department of Regenerative Medicine and Cell Biology, Medical University of South Carolina, Charleston, SC, USA; 4 School of Medical & Molecular Biosciences, University of Technology Sydney, Sydney, NSW 2000, Australia; 5 School of Biomedical Science and Pharmacy, The University of Newcastle, Callaghan, NSW 2308, Australia

## Abstract

Fibulin-1 is an extracellular matrix (ECM) protein, levels of which are elevated in serum and lung tissue from patients with idiopathic pulmonary fibrosis compared to healthy volunteers. Inhibition of fibulin-1C, one of four fibulin-1 isoforms, reduced proliferation and wound healing in human airway smooth muscle (ASM) cells. This study identified the bioactive region/s of fibulin-1C which promotes fibrosis. Seven fibulin-1C peptides were synthesized and used to pre-coat tissue culture plates before lung derived ASM cells and fibroblasts from patients with pulmonary fibrosis (PF), chronic obstructive pulmonary disease (COPD) or neither disease (Control) were plated. Peptide effects on *in vitro* measures of fibrosis: cell attachment, proliferation and viability, and ECM deposition, were examined. Among these peptides, peptide 1C1 (FBLN1C1) enhanced ASM cell and fibroblast attachment. FBLN1C1 increased mitochondrial activity and proliferation in fibroblasts. In addition, FBLN1C1 stimulated fibulin1 deposition in PF and COPD fibroblasts, and augmented fibronectin and perlecan deposition in all three groups. Peptides FBLN1C2 to FBLN1C7 had no activity. The active fibulin-1C peptide identified in this study describes a useful tool for future studies. Ongoing investigation of the role of fibulin-1 may reveal the mechanisms underlying the pathphysiology of chronic lung diseases.

Pulmonary structural remodelling is a feature of the lungs in both pulmonary fibrosis (PF) and chronic obstructive pulmonary disease (COPD)^1^,^2^,^3^,^4^. The remodelling includes alterations in the interstitial tissue, such as accumulation or destruction of extracellular matrix (ECM), and changes in the number and functions of parenchymal cells. In PF, there is an increased lung matrix deposition and proliferative and activated fibroblasts in the parenchyma^3^,^5^. In COPD, there is a destruction of the alveolar walls and interstitial tissue, termed emphysema, in the lung parenchyma^2^. However, some specific ECM proteins per weight unit are increased in the lungs of patients with emphysema compared to patients without emphysema^6^,^7^,^8^. Furthermore, peripheral airways in COPD, especially those close to emphysematous destruction, have thickened airway walls and augmented deposition of ECM^9^,^10^. The mechanisms of the development of these pathologies present in the lungs with COPD or PF are complicated. One of the remaining unanswered questions is how altered ECM proteins influence the persistence of lung remodelling in COPD and PF.

The ECM is a complex structured network of macromolecules which form the scaffold of the human lung. ECM proteins can be produced by immune and lung structural cells including epithelium, airway smooth muscle (ASM) cells and fibroblasts. However, fibroblasts are one of the major producers of ECM proteins^11^. The interaction between the ECM and the cells is dynamic, and ECM proteins can influence cellular phenotype and function^12^. Among these ECM proteins, fibulin-1 is a member of the fibulin protein family which consists of seven members (fibulin-1 to -7) in humans. Fibulin-1 is localized in the basement membrane and connective tissue in human lung and is associated with many ECM proteins to facilitate ECM functions^13^,^14^. Altered fibulin-1 levels are associated with tumour cells, chronic liver and kidney disease, diabetes and asthma^15^,^16^,^17^,^18^,^19^.

Fibulin-1(FBLN1) has four isoforms, named as FBLN1A, B, C, and D, which are splice variants possessing different C-terminal sequences. The different isoforms of fibulin-1 have variable functions. ECM FBLN1D decreases blood vessel number and increases endothelial apoptosis hence suppressing tumour growth^20^. It also decreases the invasive phenotype and tumour formation in human fibrosarcoma-derived cell lines and regulates the expression of metalloproteinases in breast cancer cells^19^,^21^. In contrast, an increased FBLN1C:FBLN1D ratio has been found in ovarian cancer cells and this increase is associated with the oestrogen receptor-α, which mediates the growth of epithelial ovarian carcinomas^22^,^23^.

Little is known about the function of FBLN1C, nor the regions which mediate its biological activity. In our previous research we have found that the level of fibulin-1 is elevated in the serum and bronchoalveolar lavage fluid of patients with asthma compared to people without asthma, and serum and tissue fibulin-1 levels are increased in the patients with IPF compared to those without lung diseases^17^,^24^. Furthermore we have found that gene silencing of FBLN1C reduced cell proliferation and wound healing of ASM cells and reduced features of lung disease in a murine model^17^. Given the important biological role of FBLN1C, the aim of this study was to identify the active part/s of the molecule and to further characterise the biological role of FBLN1C. This study was presented at the Thoracic Society of Australia & New Zealand Annual Scientific Meeting 2014^25^ and the American Thoracic Society International Conference 2014^26^.

## Results

### FBLN1C1 peptide increased the attachment of ASM cells and lung fibroblasts

**Figure 1:**
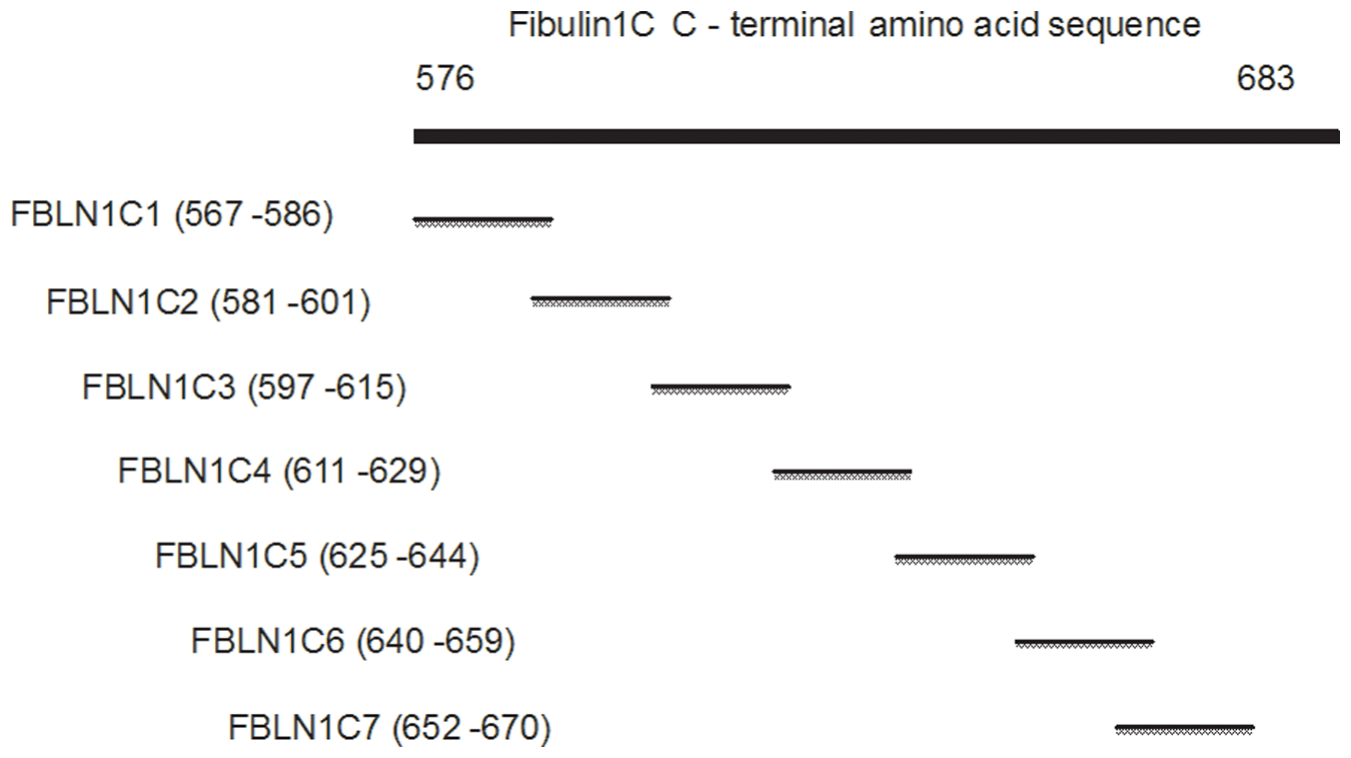
The schematic diagram of Fibulin-1C peptides. FBLN1C1-7, fibulin1C peptide 1–7.

To identify which regions of FBLN1C had biological activity, we seeded primary human lung fibroblasts and ASM cells in wells coated with seven peptides generated from FBLN1C, FBLN1C1, 2, 3, 4, 5, 6 and 7 (Fig. 1 and supplementary information Table 4), and assessed the effects on cellular attachment and viability/proliferation as measured via MTT assay. FBLN1C1 (3 and 10 μg/ml) enhanced cell attachment of both fibroblasts and ASM cells, while FBLN1C2 (3 and 10 μg/ml) increased attachment only in fibroblasts. FBLN1C3, 4, 5, 6 and 7 (3 and 10 μg/ml) did not have any effect on cell attachment either in fibroblasts or in ASM cells (Table 1 and supplementary information Table 1).

### FBLN1C1 peptide increased cell viability/proliferation of lung fibroblasts

FBLN1C1 (3 and 10 μg/ml) increased cell viability/proliferation fibroblasts (Table 2 and supplementary information Table 2). In contrast, FBLN1C2, 3, 4, 5, 6 and 7 (3 and 10 μg/ml) did not modulate viability/proliferation in lung fibroblasts. None of the FBLN1C peptides changed ASM cell viability/proliferation (Table 2 and supplementary information Table 2). Human plasma FN (hFN, 3 and 10 μg/ml) was used as a positive control in these experiments and it stimulated cell attachment and viability/proliferation of both lung fibroblasts and ASM cells (Table 1 and 2, supplementary information Table 1 and 2).

In light of the lack of activity observed with FBLIN1C2, 3, 4, 5 & 7 these peptides were not further interrogated in this study, however FBLN1C6 was maintained in the experimental setup as a negative control peptide. In all subsequent experiments we used only fibroblasts as ASM viability/proliferation was not modulated by FBLN1C1.

**Figure 2:**
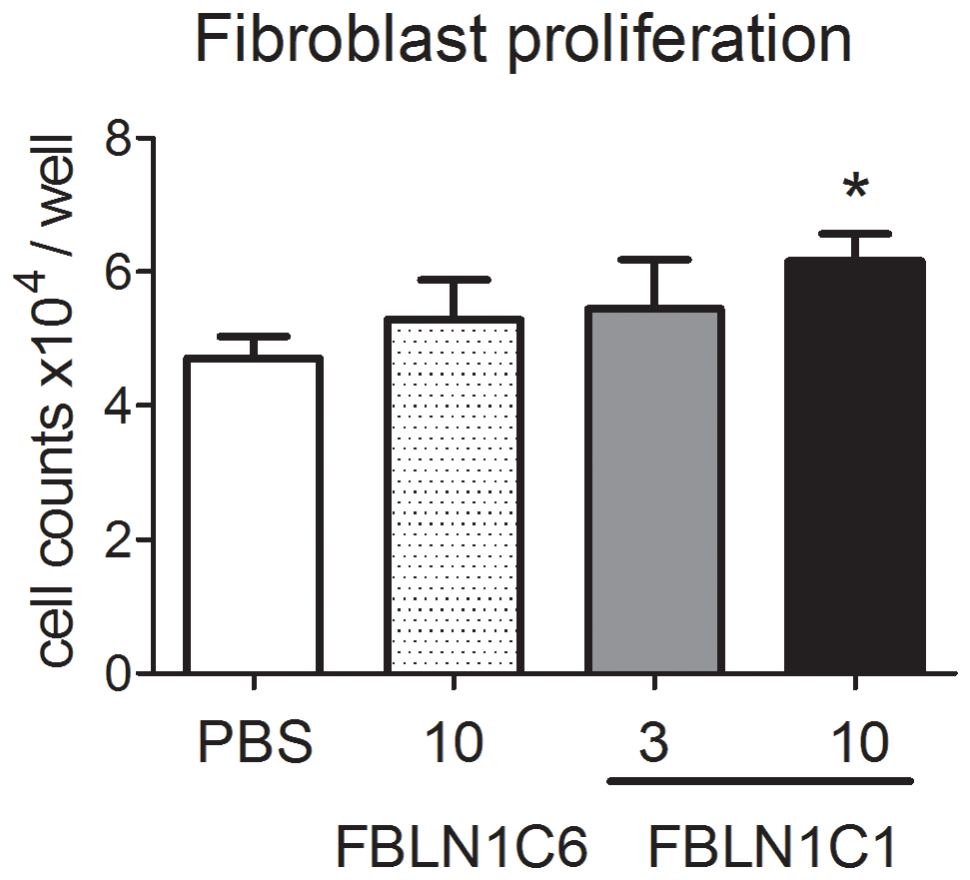
Fibulin-1C peptide 1 (FBLN1C1) induces lung fibroblast proliferation. Fibroblasts were seeded in 12-well plates, coated with FBLN1C6 (10 μg/ml, shaded bar) or FBLN1C1 (3 μg/ml, grey bar; 10 μg/ml, black bar) or neither (PBS, open bar) in 0.1% FBS DMEM and were manually counted 3 days later. N = 4, 2/4 control, 2/4 chronic obstructive pulmonary disease. * P < 0.05, significantly different from PBS; repeated One-way ANOVA with Dunnett's multiple comparison test was performed.

Since FBLN1C1 increased fibroblast viability/proliferation, we tested the effect of FBLN1C1 on fibroblast proliferation using manual cell counting. FBLN1C1 stimulated fibroblast proliferation, at a concentration of 10 μg/ml (Fig. 2) (fold increase compared to uncoated wells were 1.16 ± 0.14 and 1.32 ± 0.07, P < 0.05 for 3 μg/ml and 10 μg/ml of FBLN1C1, respectively, n = 4). The cell number was also augmented by hFN (3 μg/ml) but not FBLN1C6 (10 μg/ml, Fig. 2) (fold increase compared to uncoated wells were 1.73 ± 0.21, P < 0.05 and 1.12 ± 0.071 for hFN and FBLN1C6, respectively, n = 4). The data from these studies showed that FBLN1C1 at a concentration of 10 μg/ml was effective in promoting fibroblast attachment, viability and proliferation.

**Figure 3:**
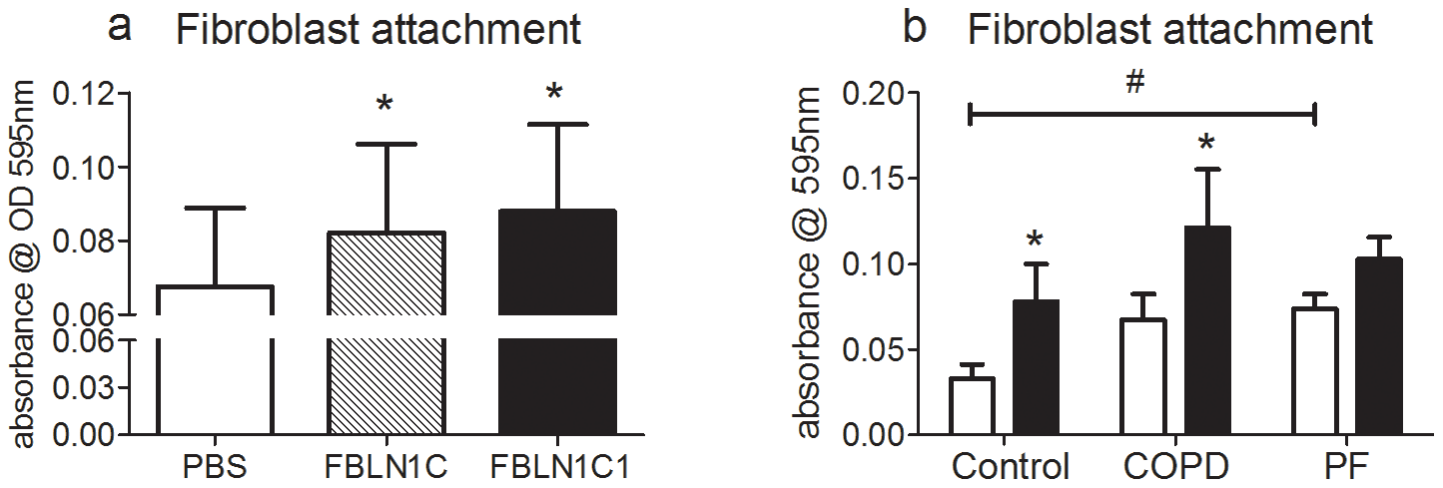
Fibulin-1C peptide 1 (FBLN1C1) and fibulin-1C (FBLN1C) protein increase lung fibroblasts attachment in control and COPD groups. Fibroblasts were seeded in 96-well plates, coated with FBLN1C protein (shaded bar) or FBLN1C1 (black bars) or neither (PBS, open bars) in 0.1% FBS DMEM. After 2 hours, cells were fixed and stained with 0.5% toludine blue solution. The dye bound to cells was solubilised using 1% SDS solution and the intensity of blue colour was detected at a wavelength of 595 nm. The results are absorbance subtracted background and expressed as mean ± SEM. (a) fibroblast attachment stimulated by fibulin-1C protein 3 μg/ml and FBLN1C1 10 μg/ml; N = 4, 2/4 are control and 2/4 are chronic obstructive pulmonary disease (COPD). (b) fibroblast attachment stimulated by FBLN1C1 10 μg/ml; Control N = 5, COPD N = 7, pulmonary fibrosis (PF) N = 8. * P < 0.05, significantly different from PBS; two-way ANOVA with Bonferroni post test was performed. # P < 0.05, significant difference between Control and PF at basal levels; one-way ANOVA with Bonferroni's multiple comparison test was performed.

To compare that the biological properties of FBLN1C1 with the full length human FBLN1C, we purified FBLN1C from HT1080 cells that were stably transfected with full length hFBLN1C plasmid to use in cell attachment assays. We found that FBLN1C protein (3 μg/ml) had similar effects as FBLN1C1 (10 μg/ml) on fibroblast attachment (Fig. 3a).

### FBLN1C1 differentially regulated cell attachment, viability and proliferation in lung fibroblasts

To further understand the biological activity of FBLN1C we investigated if it had differential effects in fibroblasts isolated from patients with lung diseases with varying amounts of fibrosis. COPD is characterised by relatively minor lung fibrosis in comparison to PF. In addition we used cells from patients that were not diagnosed with either disease (control).

Comparing the three groups of lung fibroblasts, the basal levels of cell attachment were highest in PF cells when compared to COPD and control cells (Fig. 3b, 0.033 ± 0.009, n = 5, 0.067 ± 0.015, n = 7 and 0.074 ± 0.009^#^, n = 8 for control, COPD and PF, respectively. Data are expressed as Mean ± SE of absorbance at 595 nm. ^#^, P < 0.05, one-way ANOVA, compared to control). FBLN1C1 increased fibroblast attachment in the Control and COPD groups but not in the PF group (Fig. 3b). Fibroblast attachment was enhanced by the positive control (FN, 3 μg/ml) in all three groups (Control, 508.5 ± 89.15, n = 5, P < 0.001; COPD, 323.5 ± 45.82, n = 7, P < 0.01; PF, 319.0 ± 56.54, n = 8, P < 0.001; data expressed as Mean ± SE, percentage change compared to unstimulated; supplementary information Fig. 1).

**Figure 4:**
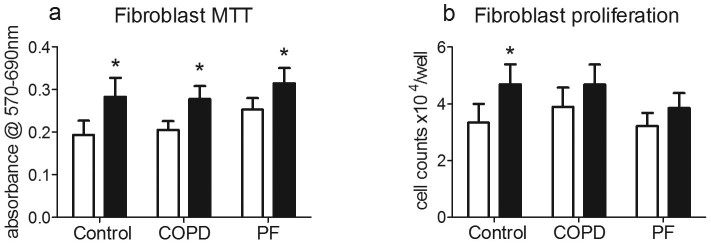
Fibulin-1C peptide 1 (FBLN1C1) increases cell viability and proliferation in lung fibroblast. Fibroblasts were seeded in the plates coated with (black bars) or without (PBS, open bars) FBLN1C1 10 μg/ml in 0.1% FBS DMEM for 3 days. (a) MTT was added into wells and converted to MTT formazan, which was dissolved using 10% SDS solution. The intensity of blue colour was detected at a wavelength of 570 nm subtracted with a reference wavelength 690 nm. The results are absorbance corrected with background and expressed as mean ± SEM. Control N = 5, chronic obstructive pulmonary disease (COPD) N = 7, pulmonary fibrosis (PF) N = 9. (b) manual cell counting for fibroblasts. The results are cell number per well and expressed as mean ± SEM. Control N = 5, COPD N = 4, PF N = 9. * P < 0.05, significantly different from PBS; two-way ANOVA with Bonferroni post test was performed.

Cell viability/proliferation under unstimulated conditions (no protein coating or proproliferative stimulus) was similar between control, COPD and PF fibroblasts. FBLN1C1 (10 μg/ml) increased viability/proliferation in all three groups (Fig. 4a), as did hFN (3 μg/ml, supplementary information Fig. 2a). However, FBLN1C1 (10 μg/ml) enhanced proliferation of the control group (Fig. 4b). Similar growth patterns were observed in fibroblasts treated with hFN (3 μg/ml) in all three groups of cells (supplementary information Fig. 2b).

### FBLN1C1 increased ECM FBLN1, EDA-FN and perlecan deposition

**Figure 5:**
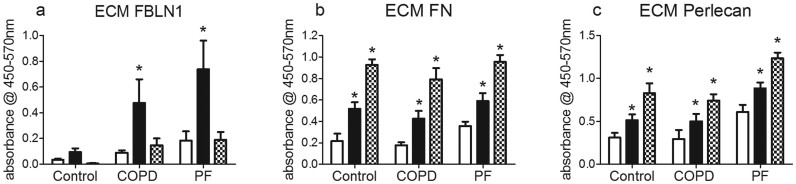
Fibulin-1C peptide 1 (FBLN1C1) increases fibulin-1, fibronectin and perlecan deposition in lung fibroblasts. Fibroblasts were seeded in 96-well plates, coated with (black bars) or without (PBS, open bars) FBLN1C1 10 μg/ml or with hFn 3 μg/ml (shaded bars), in 0.1% FBS DMEM. After 3 days, extra cellular matrix (ECM) was harvested and proteins were detected using ELISA. (a) ECM fibulin-1 (FBLN1) was determined using antibody specific for FBLN1 N-terminal. Control N = 5, chronic obstructive pulmonary disease (COPD) N = 7, pulmonary fibrosis (PF) N = 8. (b) ECM fibronectin (FN) was determined using antibody specific for extra domain A of FN. Control N = 5, COPD N = 8, PF N = 8. (c) ECM perlecan was determined using antibody specific for clone 7B5. Control N = 4, COPD N = 7, PF N = 7. The results are absorbance subtracted with background and expressed as mean ± SEM. * P < 0.05, significantly different from PBS; two-way ANOVA with Bonferroni post test was performed.

To investigate if FBLN1C1 could promote the production of ECM proteins, the deposition of FBLN1, cellular-FN and perlecan by fibroblasts was investigated. To avoid cross detection, antibodies specific for fibulin-1 N-terminal domain and EDA-FN were used in the ELISA which ensured that peptide FBLN1C1 and hFN were not detected. FBLN1C1 (10 μg/ml) stimulated FBLN1 deposition in fibroblasts from COPD and PF groups while hFN (3 μg/ml) had no effect on FBLN1 production in all three groups (Fig. 5a). Both FBLN1C1 (10 μg/ml) and hFN (3 μg/ml) increased EDA-FN and perlecan deposition in all three groups (Fig. 5b and c).

### FBLN1C1 and hFN decreased TGFβ1 release in lung fibroblasts

**Figure 6:**
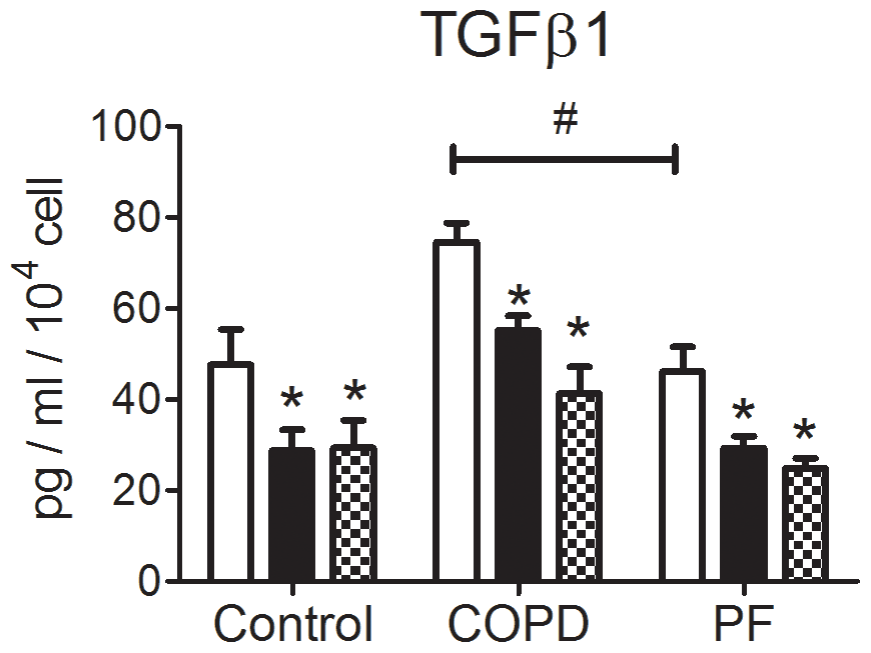
Fibulin-1C peptide 1 (FBLN1C1) decreases TGFβ1 release in lung fibroblasts. Fibroblasts were seeded in 12-well plates, coated with (black bars), without FBLN1C1 10 μg/ml (PBS, open bars) or with plasma fibronectin 3 μg/ml (shaded bars), in 0.1% FBS DMEM. After 3 days, supernatants were collected and the levels of total soluble TGFβ1 were measured using ELISA. The results are TGFβ1 concentration corrected with cell number and expressed as mean ± SEM. Control N = 5, COPD N = 4, PF N = 9. * P < 0.05, significantly different from PBS; # P < 0.05, significant difference between COPD and PF at basal levels; two-way ANOVA with Bonferroni post test was performed.

Since TGF β 1 plays a critical role in ECM regulation, we explored the possibility that FBLN1C1 induced responses in lung fibroblasts are mediated through the release of TGFβ1, a known potent pro-fibrotic stimulus for fibroblasts. Both FBLN1C1 (10 μg/ml) and hFN (3 μg/ml) decreased TGFβ1 release in all three groups (Fig. 6).

## Discussion

This study is, to our knowledge, the first to characterise the direct biological function of fibulin1C on mesenchymal cells. The active region within FBLN1C which stimulated cell attachment, viability, proliferation, and ECM protein production by lung fibroblasts was identified. FBLN1C1 differentially regulated cell proliferation and ECM FBLN1 deposition in fibroblasts from patients with or without COPD or PF.

Both lung fibroblasts and ASM cells were used in the initial experiments. The increase in cell attachment in both fibroblasts and ASM cells indicate that FBLN1C1 may have the same binding site in these two types of mesenchymal cells. The finding that FBLN1C1 enhanced fibroblast viability and proliferation indicated that the cellular signalling of FBLN1C1 may be different in fibroblasts compared to ASM cells.

The attachment of fibroblasts is a critical step in both proliferation and migration, and as such for the development of fibroblastic foci. This study demonstrated that the FBLN1C1 peptide increased cell attachment in the control and COPD groups. In the PF group, FBLN1C1 did not further enhance fibroblast attachment. This may be due to the elevated attachment levels under basal conditions in PF cells as presented in the current study.

The augmented viability and proliferation induced by FBLN1C1 in fibroblasts from all three groups indicated that FBLN1C1 has a dynamic role in fibroblast activation. However, the mechanism of this activation is unknown. hFN, the control protein used in the study, is the predominant component of FN-based fibrillar matrices, and has been found to stimulate attachment, proliferation, migration and differentiation as well as activation of inflammatory response in fibroblasts^27^,^28^. All the cells used in this study responded to hFN in terms of attachment and viability. However, there is no homology between the FBLN1C1 amino acid sequence and the hFN sequence of three amino acids or more. Therefore, it is unlikely that FBLN1C1 and hFN share the same mechanism in regulating fibroblast attachment and mitochondrial activity.

It is interesting that FBLN1C1 increased fibroblast proliferation in the control group, as did hFN. This result is consistent with the finding observed by Lau *et al.* that silencing FBLN1C decreased ASM cell proliferation^17^. Data from other studies show that the proliferation rates of fibroblasts from patients with COPD or PF are different from those from controls. However, the results from those studies are contradictory, with some reporting increased and some decreased proliferation. In addition, fibroblasts from COPD or PF lungs also have differential proliferative responses to various stimuli compared to control cells^5^,^29^,^30^. In the current study, although we did not find differences in fibroblast proliferation under basal conditions in all three groups, the increase in cell number in the presence of FBLN1C1 in only the control group illustrated that fibroblasts from the COPD and PF groups had distinct responses to FBLN1C1 with respect to cell proliferation. It is possible that the fibroblast proliferation pattern may differ when FBLN1C1 is added in the presence of other stimuli.

Mitochondria play an important role in cell growth and the MTT assay is often used as a surrogate for cell viability and proliferation assay in many cases, including in transformed fibroblast cell lines and ASM cells^31^,^32^. However, the number of mitochondria per cell is not static and is altered in respiratory diseases^33^. Thus, the increase in mitochondrial activity may not reflect cell proliferation, as we observed in the COPD and PF groups in this study.

One of the important findings in this study is the increased deposition of ECM proteins induced by FBLN1C1. In addition to cellular-FN, we also measured perlecan. Perlecan is a major heparin sulphate proteolycan in connective tissue whose function is mainly dependant on binding and presentation of growth factors to high-affinity tyrosine kinases receptors^34^. In particular, fibroblast growth factor-binding protein is a key ligand for perlecan^35^. Through this interaction, perlecan promotes functions of fibroblast growth factor and it also increases cell attachment in ASM cells from patients with COPD^36^,^37^. The augmented deposition of cellular-FN and perlecan modulated by FBLN1C1 may enhance fibroblast activity and extend fibroblast induced remodelling in the lung.

The most interesting finding of this study is that FBLN1C1 increased fibulin-1 deposition in COPD and FP groups. In contrast to cellular-FN and perlecan deposition, fibulin-1 production was not induced by hFN. This specific induction of fibulin-1 in fibroblasts in COPD and PF group indicated that FBLN1C may play a crucial role in lung remodelling in COPD and PF. We have shown that TGFβ1 was down regulated by FBLN1C1 which indicated that the FBLN1C1 induced cell attachment, proliferation and ECM deposition in lung fibroblasts is not dependent on the TGFβ1 signalling pathway. Further studies are needed to investigate the mechanisms underlying these cellular responses which may help understanding the pathogenesis of COPD and PF.

There is currently very little known information relating to FBLN1C, such as the receptors to which it binds, and an inadequacy which is not advanced by the lack of a commercially available source of FBLN1C protein for research. These limitations restrict researchers' endeavours to further investigate the functional roles of FBLN1C protein. To our knowledge, this is the first study that has investigated the activity of the specific regions of FBLN1C using peptides. The active peptide identified in this study describes a useful tool for future studies. Ongoing investigation of the role of fibulin-1 may reveal the mechanisms underlying the pathphysiology of chronic lung diseases.

## Methods

### Study subjects and primary lung cell culture

All experimental protocols using human lung tissue and information related to study subjects were approved by the Ethics Review Committee of the South West Sydney Area Health Service, the Human Research Ethics Committee of The University of Sydney and St Vincents Hospital. ASM cells and distal lung fibroblasts (fibroblasts) were obtained and maintained as previously described^38^,^39^. All methods were carried out according to guidelines set by The University of Sydney. Briefly, ASM cells were isolated from medium to large airways and fibroblasts were derived from proximal lung tissues which were obtained from macroscopically normal surgical tailings following resection for thoracic lesions, and from lung transplantation for emphysema or pulmonary fibrosis. All study subjects provided written informed consent. ASM cells and fibroblasts were maintained in Dulbecco's Modified Eagle Medium (DMEM) (Gibco, Grand Island, New York, US) with 5% foetal bovine serum (FBS) and 1% Antibiotic-Antimycotic (Gibco, Grand Island, New York, US). The cells were mycoplasma free and used in experiments between passage 4 to 7 for ASM cells and passage 2–5 for fibroblasts, respectively. The disease states of the patients were confirmed by doctor diagnosis. In this study, the cells were categorized into three groups according to doctors' diagnosis: (1) control group, cells from macroscopically normal surgical tailings of patients with lung cancer but no airway obstruction (FEV1: FVC ≥ 70%); (2) COPD group, cells from lung resection or transplantation of patients with moderate to severe airway obstruction; (3) pulmonary fibrosis (PF) group, cells from explanted lungs of patients with severe lung fibrosis. The patient demographics and which cells were used for experiments in the comparisons of the three groups are shown in supplementary information table 3.

### FBLN1C peptide design

The FBLN1C C-terminal specific region starts at amino acid 567 (NCBI Ref NP-001987.2). Seven sequential fibulin-1C specific peptides (Auspep, Tullamarine, Vic, Australia) were designed according to the principles described by Angeletti^40^. The sequences of peptides FBLN1C-1-7 are listed in supplementary information table 4. Figure 1 shows the schematic diagram of Fibulin-1C peptides which illustrates the overlapping peptide design strategy.

### Cell experiments

The plastic cell culture plates were coated with or without FBLN1C peptides at concentrations of 3 and 10 μg/ml for 20 hours (hrs) at room temperature (RT). The plates were washed three times with phosphate buffered saline (PBS) following coating and then blocked with 1% bovine serum albumin (BSA, Sigma-Aldrich, St. Louise, MO) in PBS for 1 hour (hr) at RT. After the plates were washed, ASM cells and fibroblasts were plated in DMEM containing 0.1% FBS (DMEM/FBS). 96-well plates were used for attachment, mitochondrial activity (MTT) assay and ECM deposition experiments and cells were seeded in triplicate for each treatment. 12-well plates were used for the proliferation assays. Human plasma fibronectin (hFN) (3 and 10 μg/ml, BD, Franklin Lakes, NJ) was used as positive control. Cells maintained in the culture media until the end of each experiment. The cell seeding density used in all experiments was 1 × 10^4^ cell/cm^2^ except for the attachment assay which was 5 × 10^4^ cell/cm^2^.

### Attachment assay

The attachment assay was performed as previously described^41^. Details were described in supplementary information.

### Determination of cell viability and proliferation

Cell viability and proliferation was determined using 3-[4,5-dimethylthiazol-2-yl]-2,5-diphenyl tetrazolium bromide (MTT) mitochondrial activity assay as recommended by the manufacturer (Sigma, Saint Louis, Missouri, US). Details were described in supplementary information.

### Isolation of fibulin-1C protein from conditioned media of HT1080 cells

Fibulin-1 C was purified from conditioned culture media (CCM) of HT1080 fibrosarcoma cells that were stably transfected with full length coding sequence of fibulin-1C as described by Tran et al 1997^42^. Briefly, HT1080 cells were cultured in roller bottles for 5 days in serum-free DMEM. Media was collected and passed over a sepharose CL-4B column and then over a column containing fibulin-1 antibody-conjugated sepharose. After extensive washing, fibulin-1C was eluted using 3 M KSCN buffer. Peak fractions were combined and dialyzed 2 times against 100× volume of PBS. Fibulin-1C was then passed over gelatin, heparin and wheat germ agglutinin (WGA) columns sequentially and eluted from WGA with 0.5 M of N-acetylglucosamine in tris buffered saline (TBS). Eluted fractions were dialyzed twice against 100× volume of TBS, aliquoted and stored at −20°C.

### Detection of ECM proteins using ELISA

The detection of ECM FBLN1, fibronectin (FN) and perlecan was done according to the methods described previously with some modifications^43^. Details were described in online supplement.

### Transforming growth factor (TGF) β1 ELISA

The supernatants were collected from 12-well plates after three days of treatment with FBLN1C1. The levels of total TGFβ1 in the supernatants were measured using an ELISA kit according to the manufacturer's instructions (R&D Systems, Minneapolis, MN).

### Statistical analysis

Data are expressed as mean ± SEM. Statistical analysis was performed using repeated measures one-way analysis of variance (ANOVA) and Dunnett's multiple comparison test or two-way ANOVA and Bonferroni post test analysis as appropriate (GraphPad Prism 5.0).

## Supplementary Material

Supplementary Information Supplementary information

## Figures and Tables

**Table 1:** The effect of FBLN1C peptides on cell attachment in fibroblasts and ASM cells

Cell type	Peptides	FBLN1C						hFN
	1	2	3	4	5	6	7	
Fibroblast N = 5	↑	↑	----	----	----	----	----	↑
ASM cell N = 6	↑	----	----	----	----	----	----	↑

Lung fibroblasts and airway smooth muscle (ASM) cells were seeded on procoated wells for 2 hrs. The wells were washed and stained with toluidine blue. Results are detected at 595 nm subtracted with background. N = 5 for fibroblast, 3/5 control, 2/5 chronic obstructive pulmonary disease (COPD). N = 6 for ASM cell, 4/6 control, 2/6 COPD. FBLN1C1-7, fibulin1C peptide 1-7. hFN, human fibronectin. The concentrations of FBLN1C1-7 and hFN, used for experiments, were 3 and 10 μg/ml. ↑, P < 0.05 significantly increased compared to PBS; ---- no change compared to PBS; repeated One-way ANOVA with Dunnett's multiple comparison test was performed.

**Table 2:** The effect of FBLN1C peptides on cell mitochondrial activity in fibroblasts and ASM cells

Cell type	Peptides	FBLN1C						hFN
	1	2	3	4	5	6	7	
Fibroblast N = 4	↑	----	----	----	----	----	----	↑
ASM cell N = 5	↑	----	----	----	----	----	----	↑

Lung fibroblasts and airway smooth muscle (ASM) cells were seeded on precoated wells for 3 days and incubated with MTT for 4 hrs. MTT formazan was dissolved in 10% SDS and the intensity of blue colour detected using a SpectraMax M2 plate reader. Data were collected from N = 4 for fibroblast, 3/4 control, 1/4 chronic obstructive pulmonary disease (COPD); and N = 5 for ASM cell, 4/5 control, 1/5 COPD. FBLN1C1-7, fibulin1C peptide 1-7; hFN, human fibronectin. The concentrations of FBLN1C1-7 and hFN, used for experiments, were 3 and 10 μg/ml. ↑, P < 0.05 significantly different compared to PBS; ---- no change compared to PBS; repeated One-way ANOVA with Dunnett's multiple comparison test was performed.
