# Supplementary material for: Fibulin1C peptide induces cell attachment and extracellular matrix deposition in lung fibroblasts
Source: Sci Rep. 2015 Apr 2;5:9496. doi: 10.1038/srep09496 (PMC5381689; doi:10.1038/srep09496)
Supplement: Supplementary Information [file srep09496-s1.pdf]

Fibulin1C peptide induces cell attachment and extracellular matrix deposition in lung fibroblasts

Qi Ge[a, b*], Ling Chen[a], Jade Jaffar[a], William Scott Argraves[c], Waleed O. Twal[c], Phil Hansbro[e], Judith L. Black[a, b], Janette K. Burgess[a, b#] and Brian Oliver[a, b,d#]

[a] Respiratory Cellular and Molecular Biology Group, Woolcock Institute of Medical Research, Sydney, NSW 2037, Australia;

[b] Discipline of Pharmacology, Sydney Medical School, The University of Sydney, NSW 2006, Australia;

[c] Department of Regenerative Medicine and Cell Biology, Medical University of South Carolina, Charleston, SC, USA.

[d] School of Medical & Molecular Biosciences, University of Technology Sydney, Sydney, NSW 2000, Australia.

[e] School of Biomedical Science and Pharmacy, The University of Newcastle, Callaghan, NSW 2308, Australia.

[#] These authors made equal contributions.

Corresponding author: Qi Ge
Woolcock Institute of Medical Research
431 Glebe Point Road, Glebe
NSW 2037 Australia
Phone: +61 2 91140357
Fax: +61 2 91140399
E-mail: qi.ge@sydney.edu.au

Supplementary information:

Results

Table 1 The effect of FBLN1C peptides on cell attachment in fibroblasts and ASM cells

	Fibroblast N=5			ASM cell N=6		
Conc<br><br>Peptide	PBS	3 µg/ml	10 µg/ml	PBS	3 µg/ml	10 µg/ml
FBLN1C1	100	150.2±13.2*	131.7±12.1*	100	174.1±12.4*	154.2±12.4*
FBLN1C2	100	141.1±15.0*	151.4±19.4*	100	123.7±8.8	110.9±20.8
FBLN1C3	100	94.7±3.1	86.7±6.3	100	107.4±14.9	112.0±6.0
FBLN1C4	100	96.1±8.6	91.3±4.8	100	102.4±8.8	124.5±8.9
FBLN1C5	100	92.5±7.3	82.5±6.6	100	108.0±9.3	104.6±13.2
FBLN1C6	100	102.7±4.5	114.2±18.8	100	105.2±11.9	105.1±15.3
FBLN1C7	100	109.8±5.9	91.3±6.9	100	97.0±11.4	109.7±15.1
hFN	100	241.1±21.9*	248.1±26.6*	100	312.5±68.6*	345.0±71.1*

Lung fibroblasts and airway smooth muscle (ASM) cells were seeded on precoated wells for 2 hrs. The wells were washed and stained with toluidine blue. Data are absorbance at 595nm less background and expressed as percentage of PBS (Mean ± SE). N = 5 for fibroblast, 3/5 control, 2/5 chronic obstructive pulmonary disease (COPD). N = 6 for ASM cell, 4/6 control, 2/6 COPD. FBLN1C1-7, fibulin1C peptide 1-7; hFN, human fibronectin; Conc, concentration. *, $P < 0.05$ significantly different from PBS; repeated One-way ANOVA with Dunnett's multiple comparison test was performed.

Table 2 The effect of FBLN1C peptides on cell mitochondrial activity in fibroblasts and ASM cells

Cell type	Fibroblast N = 4			ASM cell N = 5		
Conc<br><br>Peptide	PBS	3 µg/ml	10 µg/ml	PBS	3 µg/ml	10 µg/ml
FNLN1C1	100	109.3±2.9*	108.3±2.9*	100	103.2±4.0	102.5±4.5
FBLN1C2	100	99.0±2.5	96.6±3.4	100	106.0±2.8	101.1±1.9
FBLN1C3	100	101.7±2.5	98.9±2.9	100	98.3±2.8	95.5±2.9
FBLN1C4	100	97.3±0.6	99.1±2.2	100	101.1±1.8	100.7±2.2
FBLN1C5	100	100.8±2.1	104.4±4.1	100	94.7±1.5	94.7±2.9
FBLN1C6	100	100.2±1.8	104.0±1.7	100	99.9±1.2	99.0±1.8
FBLN1C7	100	102.4±2.0	101.0±3.0	100	95.2±1.8	93.4±1.3
hFN	100	114.6±8.5	123.6±8.7*	100	122.8±4.7*	124.5±4.4*

Lung fibroblasts and airway smooth muscle (ASM) cells were seeded on precoated wells for 3 days and incubated with MTT for 4 hrs. MTT formazan were dissolved in 10% SDS and

the intensity of blue colour was detected using a SpectraMax M2 plate reader. Data are absorbance and expressed as percentage of PBS (Mean ± SE). N = 4 for fibroblast, 3/4 control, 1/4 chronic obstructive pulmonary disease (COPD). N = 5 for ASM cell, 4/5 control, 1/5 COPD. FBLN1C1-7, fibulin1C peptide 1-7; hFN, human fibronectin; Conc, concentration. *, $P < 0.05$ significantly different from PBS; repeated One-way ANOVA with Dunnett's multiple comparison test was performed.

Figure 1

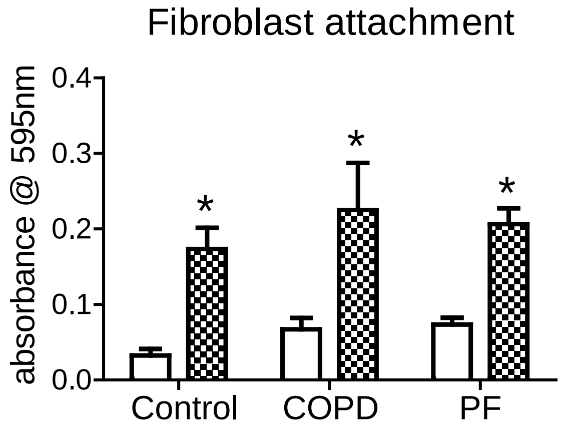

Fig. 1 Plasma fibronectin increases lung fibroblast attachment. Fibroblasts were seeded in 96-well plates, coated with (shaded bar) or without plasma fibronectin (PBS, open bars) at 3 µg/ml in 0.1% FBS DMEM. After 2 hours, cells were fixed and stained with 0.5% toludine blue solution. The dye bound to cells was solubilised using 1% SDS solution and the intensity of blue colour was detected at a wavelength of 595 nm. The results are absorbance less background and expressed as mean ± SEM. Control N = 5, chronic obstructive pulmonary disease (COPD) N = 7, pulmonary fibrosis (PF) N = 8. * $P < 0.05$, significantly different from PBS; two-way ANOVA with Bonferroni post test was performed.

Figure 2

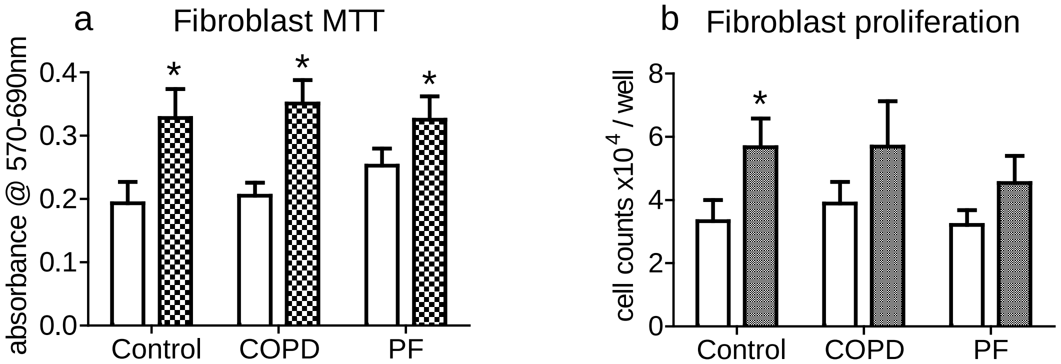

Fig. 2 Plasma fibronectin increases lung fibroblast viability and proliferation. Fibroblasts were seeded in the plates coated with (shaded bars) or without (PBS, open bars) plasma fibronectin at 3 µg/ml in 0.1% FBS DMEM for 3 days. a) MTT was added into wells and converted to MTT formazan, which was dissolved using 10% SDS solution. The intensity of blue colour was detected using the SpectraMax M2 plate reader. The results are absorbance and expressed as mean ± SEM. Control N = 5, chronic obstructive pulmonary disease (COPD) N = 7, pulmonary fibrosis (PF) N = 9. b) manual cell counting was performed. The results are cell number per well and expressed as mean ± SEM. Control N = 5, COPD N = 4, PF N = 9.
* P < 0.05, significantly different from PBS; two-way ANOVA with Bonferroni post test was performed.

Methods

Attachment assay

The attachment assay was performed as previously described [30]. Briefly, the cells were plated in precoated wells in 0.1% FBS DMEM for 2 hrs and washed with PBS after the incubation before being fixed with 4% paraformaldehyde for 10 minutes and stained with 0.5% toluidine blue solution for 5 minutes. After removal of excess dye, stained cells were washed and 1% sodium dodecyl sulphate (SDS) solution was added to release the dye. The amount of released dye was quantified using a SpectraMax M2 microplate reader (Molecular Devices, Sunnyvale, California, US) at absorbance of 595 nm. Dye absorbance readings correlate with the number of attached cells per well. Triplicate values were averaged and subtracted from background, and the treatment means were analysed for statistical differences and graphed using GraphPad Prism (Version 5.0, La Jolla, California, US).

Determination of cell viability and proliferation

Cell viability and proliferation was determined using 3-[4,5-dimethylthiazol-2-yl]-2,5-diphenyl tetrazolium bromide (MTT) mitochondrial activity assay as recommended by the manufacturer (Sigma, Saint Louis, Missouri, US). Briefly, cells were incubated for 3 days in DMEM/FBS and 4 hrs before the termination of the experiments, 10µl of 5mg/ml MTT reagent was added to each well. After 4 hrs, 100µl of 10% SDS with 0.01N HCl was then added to each well. The plates were left at 37°C overnight to dissolve the formazan. The absorbance was measured at a wavelength of 570 nm, and background absorbance was measured at 690 nm using the SpectraMax M2 plate reader. The differences of between the

primary and background absorbance for each treatment were averaged and GraphPad Prism was used for statistical analysis and graphing of the experimental means.

Detection of ECM proteins using ELISA

The detection of ECM FBLN1, fibronectin (FN) and perlecan was done according to the methods described previously with some modifications [31, 32]. Briefly, fibroblasts were cultured for 3 days, lysed with 0.016M NH_4OH and washed with PBS. The plates coated with the deposited ECM proteins were stored at -20^oC until further analysis. A mouse monoclonal anti-human Fibulin-1 antibody (Santa Cruz Biotechnology, Santa Cruz, CA, US) and a mouse monoclonal anti-human perlecan antibody (clone 7B5, Invitrogen, Camarillo, CA, US) were used as primary antibodies followed by a biotinylated chicken anti-mouse antibody (Santa Cruz Biotechnology). The FBLN1 antibody interacted with amino acids 1-190 of human FBLN1. Therefore it would not bind to any of the specific peptides used in this study. An antibody which bound to the alternatively spliced domain A (EDA) of cellular FN (EDA-FN, 1:2000 dilution, clone DH1, Merk Millipore, Billerrca, MA, US) was used to quantify fibroblast derived FN. This EDA-FN antibody did not recognize the hFN, which lacks the EDA domain, used to coat the plates. A horseradish peroxidase (HRP) conjugated streptavidin (R&D Systems, Minneapolis, MN, US) or rabbit anti mouse HRP-conjugated antibody (Dako, Glostrup, Denmark) were used as secondary antibodies. A HRP substrate 3, 3', 5, 5''-tetramethylbenzidine (Invitrogen Corporation, Camarillo, CA, US) was used to detect levels of each ECM protein. The absorbance of at 450nm wavelength was determined using the SpectraMax M2 plate reader. The readings of the wells were subtracted from control wells that contained no cells. The data were averaged from triplicate readings.

Table 3 Patient demographics

	Control	COPD	PF
Age	60.7±2.55 (N = 7)	60.4±1.87 (N = 12)	54.9±2.30 (N = 11)
FEV1 (% of predicted)	84.1±5.46 (N = 7)	38.4±7.21* (N = 12)	48.0±4.45* (N = 11)
FEV1:FVC	76.1±1.75 (N = 7)	34.0±4.63* (N = 12)	85.0±1.81** (N =11)
Smoking history<br><br><br>Pack years	6 /7 ex-smoker<br>1 /7 never smoke<br><br>41.6±4.64 (N = 5)	12 /12 ex-smoker<br>44.1±5.41 (N = 12)	7 /12 ex-smoker<br>3 / 12 never smoke<br>2 / 12 unknown<br>11.0±4.89 (N = 5)

FEV1, forced expiratory volume in 1 second. FVC, forced vital capacity. COPD, chronic obstructive pulmonary disease. PF, pulmonary fibrosis. Data are expressed as Mean ±

standard error of deviation (SE). * P < 0.05, significantly different from control; ** P < 0.05 significantly different from COPD. Krusal-Wallis test with Dunn's multiple comparison test was performed.

Table 4 The sequence of fibulin-1C peptides

Name of peptide	Sequence
FBLN1C1	RCERLPCHENRECSKLPLRI-OH
FBLN1C2	KLPLRITYYHLSFPTNIQAPA-OH
FBLN1C3	IQAPAVVFRMGPSSAVPGD-OH
FBLN1C4	AVPGDSMQLAITGGNEEGF-OH
FBLN1C5	NEEGFFTTRKVSPHSGVVAL-OH
FBLN1C6	GVVALTKPVPEPRDLLLTVK-OH
FBLN1C7	RDLLLTVKMDLSRHGTVSS-OH

FBLN1C1-7, fibulin1C peptide 1-7.
